# MoTe_2_ Polymorphs: A DFT Approach to Structural, Electronic, Mechanical and Vibrational Properties

**DOI:** 10.1021/acsomega.5c00226

**Published:** 2025-03-24

**Authors:** Lathifa Banu S, Kanimozhi Balakrishnan, Vasu Veerapandy, Nalini Vajeeston, Ponniah Vajeeston

**Affiliations:** † Department of Physics, Sethu Institute of Technology, Kariyapatti, Virudhunagar, Tamil Nadu 626115, India; ‡ Department of Computational Physics, School of Physics, Madurai Kamaraj University, Palkalai Nagar, Madurai , Tamil Nadu 625021, India; § Department of Chemistry, Center for Materials Science and Nanotechnology, University of Oslo, 0371 Oslo, Norway

## Abstract

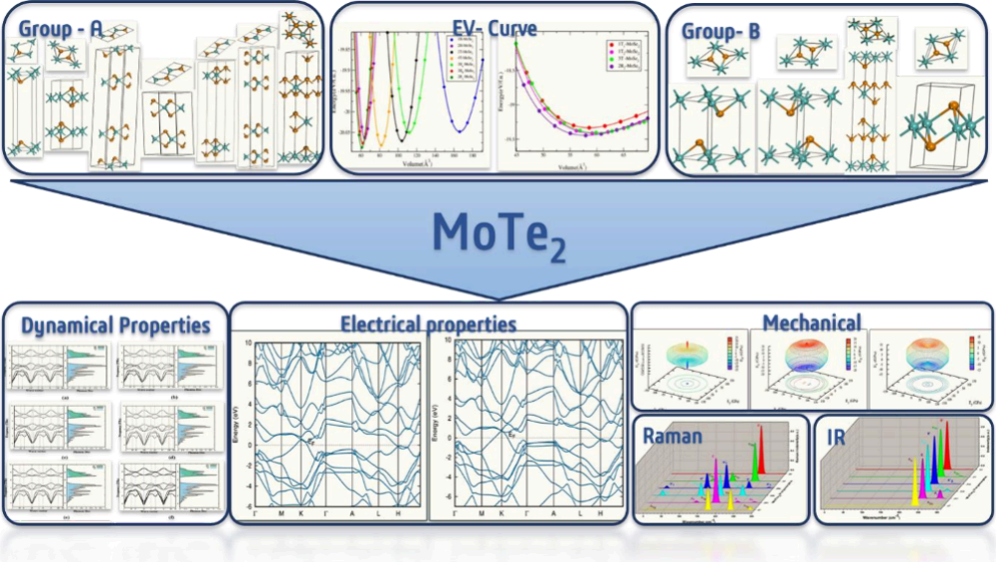

Molybdenum ditelluride (MoTe_2_), a key member of the transition metal dichalcogenides (TMDCs) family, holds significant potential for applications in electronics, energy storage, and catalysis. Despite its importance, the range of MoTe_2_ structural forms that has been explored is still limited. The primary aim of this research is to identify new stable MoTe_2_ polymorphs that may exist under zero-temperature and zero-pressure conditions. This study offers an in-depth analysis of 11 different structural variations (polymorphs) of MoTe_2_ using advanced computational methods based on density functional theory (DFT). By employing the Heyd-Scuseria-Ernzerhof (HSE06) hybrid functional, accurate calculations of electronic properties, such as band structure, are achieved. Bonding analysis, including charge density and electron localization, reveals consistent covalent interactions across the hexagonal and trigonal forms of MoTe_2_. The study also assesses the mechanical stability of these polymorphs using elastic constants, identifying both stable and metastable forms. Additionally, phonon and thermal properties, including heat capacity and entropy, are calculated for all dynamically stable polymorphs. Raman and infrared spectra provide insights into their distinct vibrational modes. These findings help distinguish structural attributes relevant to layer-specific applications. This comprehensive investigation of MoTe_2_ polymorphs uncovers new stable structures and provides crucial insights for their potential use in technological applications.

## 1 Introduction

TMDCs are a significant type of materials characterized by unique properties such as ultrathin materials for electronics, high-performance semiconductors flexible and lightweight building materials sustainable building materials, functional materials for sensors, and so on.^1^ MoTe_2_ occurs in various polymorphs, mainly including the hexagonal 2H, monoclinic 1T’, and orthorhombic Td phases.^2^ Each polymorph displays unique electronic properties: the 2H phase is a semiconductor with a moderate bandgap, making it suitable for transistors and photodetectors, whereas the 1T’ and Td phases are metallic, offering potential for topological and quantum spin Hall applications.^3^ The polymorphic versatility of MoTe_2_ allows it to transition between various electronic states when subjected to external influences (such as temperature, strain, or chemical doping), creating opportunities for phase engineering in functional devices. A comprehensive investigation of MoTe_2_ polymorphs is still lacking, despite growing interest. Some researchers have experimentally studied MoTe_2_ and their findings are summarized in the literature review as follows: An experimental study by Zhou et al., reported the impact of defects on the phase stability of MoTe_2_. Defects can potentially drive phase transitions between the semiconducting 2H phase and the metallic 1T’ or Td phases, indicating their importance in phase engineering efforts to create functional devices.^4^ Keum et al., investigated the phase transition between 2H and 1T’ phases under controlled strain, revealing electronic band structure changes theoretically.^5^ Chen et al., describe how chemical functionalization stabilizes the metastable 1T’ phase of MoTe_2_, contributing to phase control for electronic applications.^6^

Song et al., analyzed an experimental study of semimetal MoTe_2_ as multimode transport behavior.^7^ Huang et al., used the first-principles method to explain the phase transition between 2H-MoTe_2_ and 1T-MoTe_2_ even if just a few polymorphs are explored in MoTe_2_.^8^ Furthermore, Duerloo et al., explained the structural phase change in the monolayer of electrostatically doped MoTe_2_.^9^ According to Cao. et al., based on the first principle calculation, hexagonal structures can be used as photocatalysts in water-splitting applications.^10^ Naturally, the environmental changes defect the activated molecular interaction changes in MoTe_2_ which were explained by Chen et al. using the DFT method.^11^ Additionally, Yuan et al., explained that the ferroelectricity in 1T-MoTe_2_ will be down to the atomic monolayer at room temperature experimentally and theoretically.^12^ Monolayer MoTe_2_–H exhibits ultrafast band gap renormalization and optical gain accumulation was reported by theoretically Meckbach et al.^13^ These studies have collectively contributed to understanding MoTe_2_ polymorph synthesis, stability, and electronic, optical, and mechanical properties, paving the way for their potential application in new material technologies. MoTe_2_ is used for a variety of purposes; however, there is still a lack of diverse polymorph utilization for a broad range of applications. Selecting input structures for MoTe_2_ from the ICSD and Materials Project databases for the AX_2_ composition requires significant effort and computational resources.^8^ By employing energy-volume curve fitting, low-energy polymorphs are effectively identified, ultimately selecting 11 distinct MoTe_2_ polymorphs from the available options. This work primarily focused on a few known phases and unreported phases of MoTe_2_, and the investigation delves into the relative stability, electronic structure, vibrational properties, thermal behavior, and the effective masses of electrons and holes in the semiconductive polymorphs. This study provides a detailed characterization of the bonding nature in hexagonal and trigonal polymorphs through charge density, charge transfer, and electron localization function (ELF) computations. The dynamical stability of the MoTe_2_ polymorphs was conducted from phonon analyses, which were subsequently used for vibrational studies through the computation of IR and Raman spectra. A key innovative aspect of this work is the evaluation of the thermal stability of dynamically stable polymorphs, which provides valuable insights into their potential practical applications. Through this systematic exploration, we have discovered novel stable polymorphs that have been previously unreported. These newly identified stable structures opened new opportunities for their use in various applications, such as energy storage, catalysis, optoelectronics, and microelectronics.

## 2 Result and Discussion

### 2.1 Structure and the Stability of MoTe_2_ Polymorphs

MoTe_2_ features a unique structure where a molybdenum (Mo) atom is nestled between two layers of tellurium (Te), creating a Te–Mo–Te sandwich-like configuration. The combination of strong intralayer covalent bonds and weak interlayer van der Waals forces gives MoTe_2_ its unique mechanical and electronic characteristics.^14^ Within the layer, MoTe_2_ will have either a trigonal prismatic phase with a hexagonal structure or an octahedral prismatic phase with a trigonal structure. Depending on the layer stacking, MoTe_2_ can create various numbers of polymorphs. Variations in the stacking order and registration of consecutive Se–Mo–Se sandwiches of the trigonal prismatic and octahedral prismatic forms 11 different polymorphs 1H-MoTe_2_, 2H-MoTe_2_, 3H_a_-MoTe_2_, 3H_b_-MoTe_2_, 2T-MoTe_2_, 4T-MoTe_2_, 2R_1_-MoTe_2_, 1T_1_-MoTe_2_, 1T_2_-MoTe_2_, 3T- MoTe_2_ and 2R_2_-MoTe_2_, are used as the base for this investigation. In this structure, the number of layers per unit cell along the *c*-axis is defined as an integer. The structural symmetries are indicated by the letters T, H, and R representing trigonal, hexagonal, and rhombohedral symmetries, respectively. The a and b suffixes of the 3H_a_-MoTe_2_ and 3H_b_-MoTe_2_ denote the stacking arrangement of layers.

**Figure 1:**
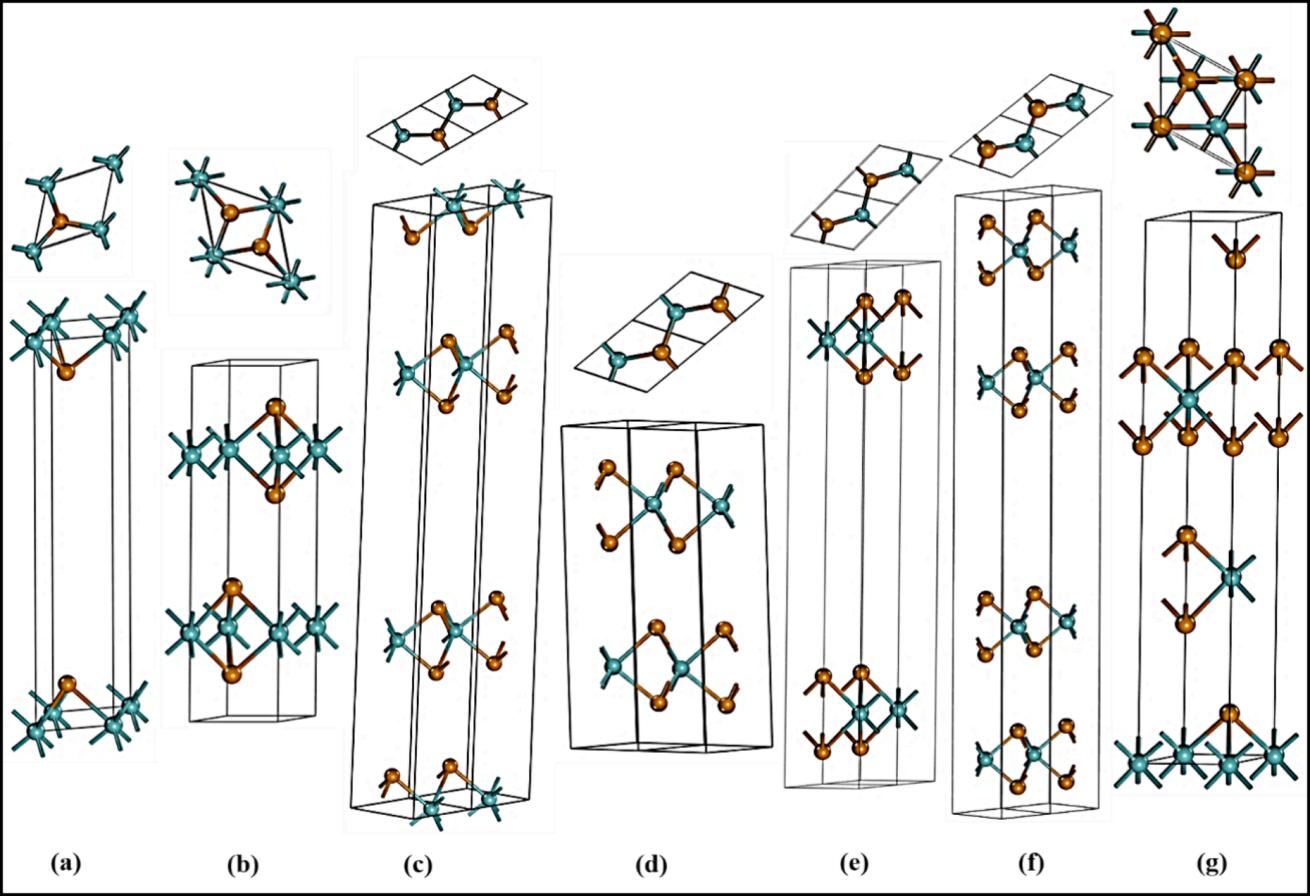
Structure and the top view of group A polymorphs are shown. 1H-MoTe_2_ in (a), 2H-MoTe_2_ in (b), 2T-MoTe_2_ in (c), 4T-MoTe_2_ in (d), 3H_a_-MoTe_2_ in (e), 3H_b_- MoTe_2_ in (f) and 2R_1_-MoTe_2_ in (g).

**Figure 2:**
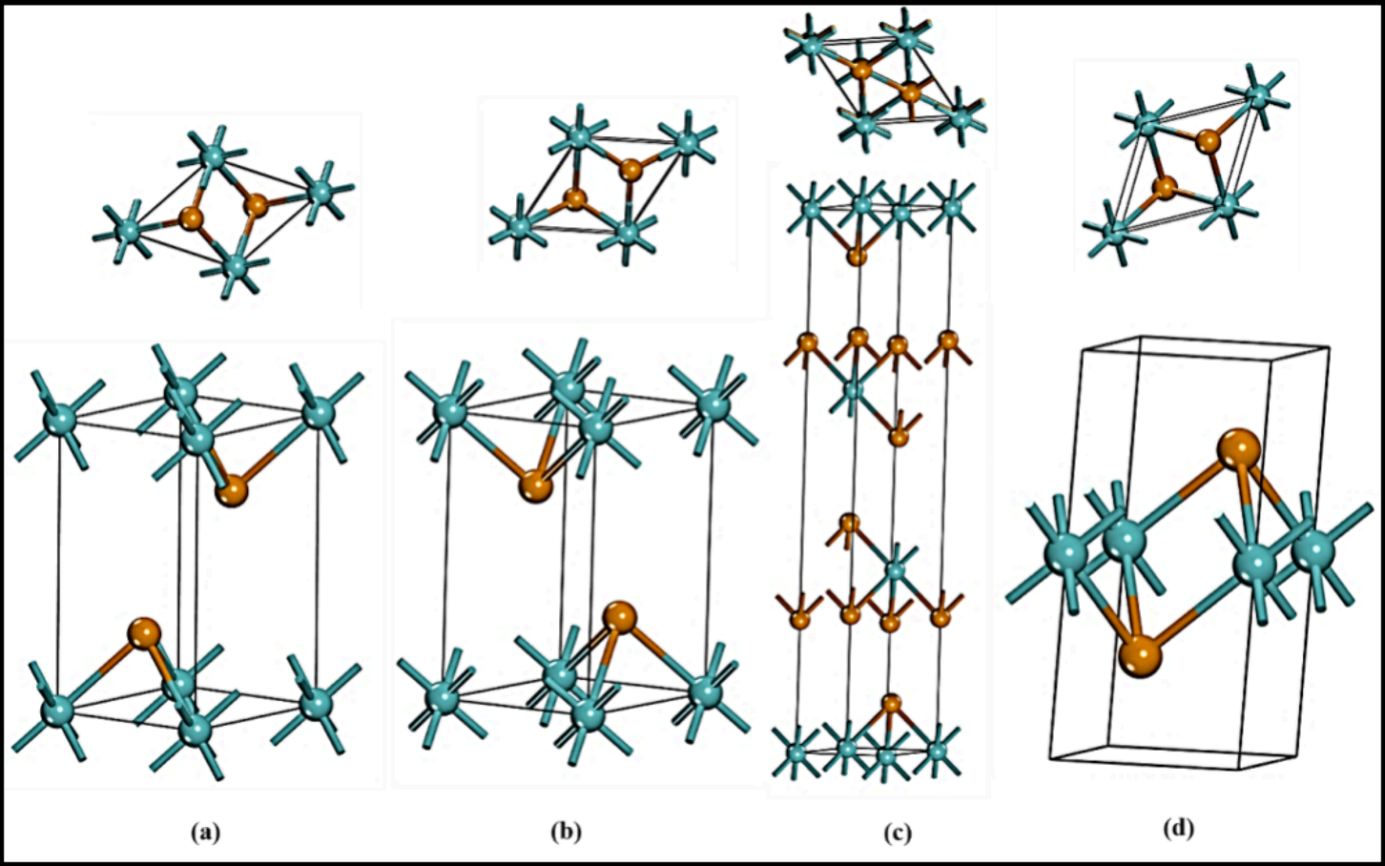
Structure and top view of group B polymorphs. 1T_1_-MoTe_2_ in (a), 1T_2_-MoTe_2_ in (b), 3T- MoTe_2_ in (c), and 2R_2_-MoTe_2_ in (d).

The total energy is determined as a function of the volume for all of the polymorphs of MoTe_2_. In this study, the polymorphs are categorized into two distinct groups, group A and group B, based on their total energy. Group A consists of the polymorphs 1H-MoTe_2_, 2H-MoTe_2_, 3H_a_-MoTe_2_, 3H_b_-MoTe_2_, 2T-MoTe_2_, 4T-MoTe_2,_ and 2R_1_-MoTe_2_ which exhibit hexagonal, trigonal, and rhombohedral structures, as illustrated in Figure 1. Group B, on the other hand, includes the polymorphs 1T_1_-MoTe_2_, 1T_2_-MoTe_2_, 3T-MoTe_2_, and 2R_2_-MoTe_2_ featuring octahedral prismatic phases as well as trigonal and rhombohedral structures, as shown in Figure 2.

**Figure 3:**
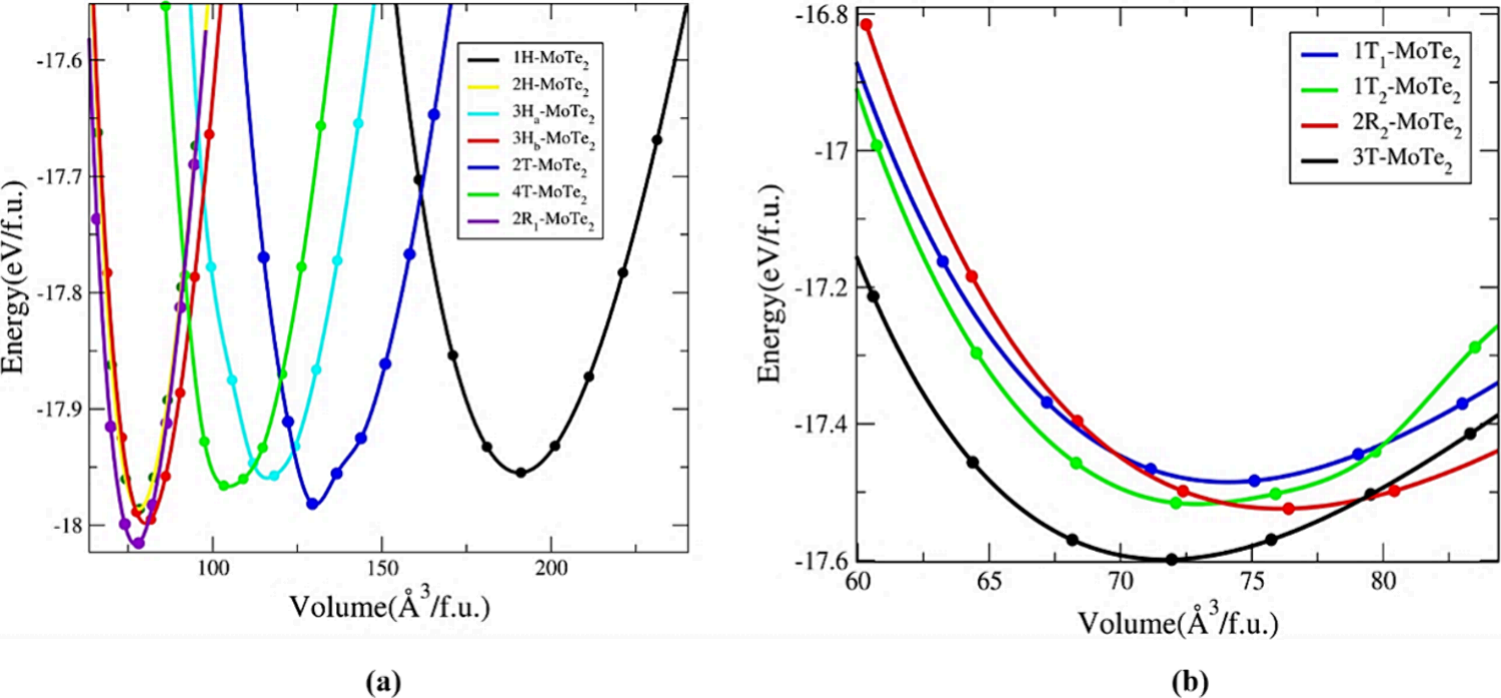
Total energy vs volume curve for group A (a) and group B (b) MoTe_2_ polymorphs. All of the energy volumes are normalized to one formula unit (f.u.).

Figure 3a,b depicts the total energy of the group A and group B polymorphs as a function of volume. Group A exhibits relatively low energy values, ranging from −18.25 to −17.95 eV/f.u. Within this group, the 2R_1_-MoTe_2_ polymorph has the lowest total energy, indicating its high stability among the polymorphs studied. Interestingly, despite their differences in volume, the 1H-MoTe_2_, 3H_a_-MoTe_2_, and 4T-MoTe_2_ polymorphs show closely aligned energy values, suggesting that their energy landscapes are quite similar. This proximity in energy indicates that these polymorphs can transition between phases with minimal energy barriers, highlighting their potential for phase flexibility and tunability in various applications. The group B polymorphs exhibit relatively low energy values, ranging from −17.6 to −17.5 eV/f.u. with the 3T-MoTe_2_ polymorph having the lowest total energy within this group. In comparison, the group A polymorphs have a lower total energy, indicating that they are more stable than those in group B. The minimum energy and corresponding volume of each polymorph are provided in Table S1 of the Supporting Information on page S1.

Initially, all of the polymorphs were optimized, and their positions along with lattice constants are summarized in Table 1. The lattice constant of the 2H-MoTe_2_ (*a* = 3.504 Å, *c* = 14.745 Å) and 1T_1_-MoTe_2_ (*a* = 3.479 Å, *c* = 7.164 Å) well coincided with the experimental and theoretical results which are shown in Table 1.^8,15^

**Table 1:** List of Calculated Unit Cell Constants and Coordinates for the Investigated MoTe_2_ Polymorphs^a^

polymorph	cell constants (Å)	coordinates				
1H-MoTe2 (*P*6̅*m*2; mp-1023924)	*a* = 3.497, *c* = 18.033	Mo1	(1a)	0.000	0.000	0.000
		Te1	(2*h*)	0.333	0.666	0.100
2H-MoTe_2_ (*P6*_*3*_*/mmc;* mp-1018809)	*a* = 3.504, *c* = 14.745	Mo1	(2*b*)	0.000	0.000	0.250
	*a* = 3.590, *c* = 13.966^9^	Te1	(4*f*)	0.666	0.333	0.372
	*a* = 3.519, *c* = 13.970^15^					
	*a* = 3.517, *c* = 13.949^16^					
3H_a_-MoTe_2_ (*P*6̅*m2;* mp-1025874)	*a* = 3.506, *c* = 33.297	Mo1	(2*g*)	0.000	0.000	0.721
		Mo2	(1*c*)	0.333	0.666	0.000
		Te1	(2*g*)	0.000	0.000	0.054
		Te2	(2*h*)	0.333	0.666	0.333
		Te3	(2*h*)	0.333	0.666	0.775
3H_b_-MoTe_2_ (*P6*_*3*_*/mmc*; mp-2815)	*a* = 3.506, *c* = 15.361	Mo1	(2*d*)	0.666	0.333	0.250
		Te1	(4*f*)	0.666	0.250	0.867
2T - MoTe_2_ (*P*3̅*m1*; mp-1023939)	*a* = 3.445, *c* = 25.180	Mo1	(2*d*)	0.666	0.333	0.859
		Te1	(2*d*)	0.666	0.333	0.212
		Te2	(2*d*)	0.666	0.333	0.067
4T- MoTe_2_ (*P*3̅*m1*; mp-1027525)	*a* = 3.269, *c* = 35.382	Mo1	(1*a*)	0.333	0.666	0.088
		Mo2	(1*a*)	0.666	0.333	0.911
		Mo3	(1*a*)	0.333	0.666	0.697
		Mo4	(1*a*)	0.666	0.333	0.302
		Te1	(1*a*)	0.333	0.666	0.347
		Te2	(1*a*)	0.666	0.333	0.652
		Te3	(1*a*)	0.333	0.666	0.957
		Te4	(1*a*)	0.666	0.333	0.042
		Te5	(1*a*)	0.333	0.666	0.743
		Te6	(1*a*)	0.666	0.333	0.866
		Te7	(1*a*)	0.333	0.666	0.133
		Te8	(1*a*)	0.666	0.333	0.256
2R_1_-MoTe_2_ (*R*3̅*m*; ICSD_31067;1434)	*a* = 3.501, *c* = 22.070	Mo1	(1*a*)	0.999	0.999	0.999
		Te1	(1*a*)	0.415	0.415	0.415
		Te2	(1a)	0.251	0.251	0.259
1T_1_-MoTe_2_ (*P*3̅*m1*; mp-147)	*a* = 3.479, *c* = 7.164	Mo1	(1*a*)	0.000	0.000	0.000
	*a* = 3.493, *c* = 6.048^8^	Te1	(2d)	0.666	0.333	0.258
1T_2_-MoTe_2_ (*P*3̅*;* mp-164)	*a* = 3.494, *c* = 6.818	Mo1	(1*a*)	0.000	0.000	0.000
		Te1	(2*d*)	0.666	0.333	0.267
3T-MoTe_2_ (*R*3̅*m*; mp-1558544)	*a* = 3.442, *c* = 21.034	Mo1	(1*b*)	0.500	0.500	0.500
		Te1	Te1	0.744	0.744	0.744
2R_2_-MoTe_2_ (*P3m1;* mp-11238797)	*a* = 3.440, *c* = 7.415	Mo1	(1*b*)	0.000	0.000	0.500
		Te1	(2*d*)	0.666	0.333	0.238

a The space group and material project ID are given near the polymorphs.

### 2.2 Electronic Structure

The electronic structures of MoTe_2_ are highly dependent on its polymorphic form. In this study, a comprehensive exploration of the electronic structure of MoTe_2_ using DFT is presented. However, standard DFT calculations using the local density approximation (LDA) are known to underestimate the band gap of semiconducting materials due to the lack of accurate treatment of electronic correlation.^17^ To address this limitation, hybrid functionals such as the Heyd-Scuseria-Ernzerhof (HSE06) are employed in our calculations to provide more reliable bandgap predictions.^18^ The band structures of both group A and group B polymorphs are calculated at zero pressure, focusing on the high-symmetry points within the first Brillouin zone.^19^ Excluding the 4T-MoTe_2_ polymorph, all of the group A polymorphs show semiconducting properties. On the other hand, the electronic structure of all the polymorphs in group B shows metallic behavior. For monolayer 1H-MoTe_2_, DFT studies indicate a direct bandgap of 1.59 eV at the *K*-point of the Brillouin zone.

Similarly, the electronic band structure of 3H_a_-MoTe_2_ exhibits a direct bandgap, with strong contributions from Mo *d*-orbitals and Te *p*-orbitals. Other polymorphs, such as 2H-MoTe_2_, 3H_b_-MoTe_2_, 2T-MoTe_2_, and 2R_1_-MoTe_2_, exhibit an indirect band gap. This band gap is sensitive to factors such as strain, external electric fields, and the number of layers, offering tunability for specific applications.

**Figure 4:**
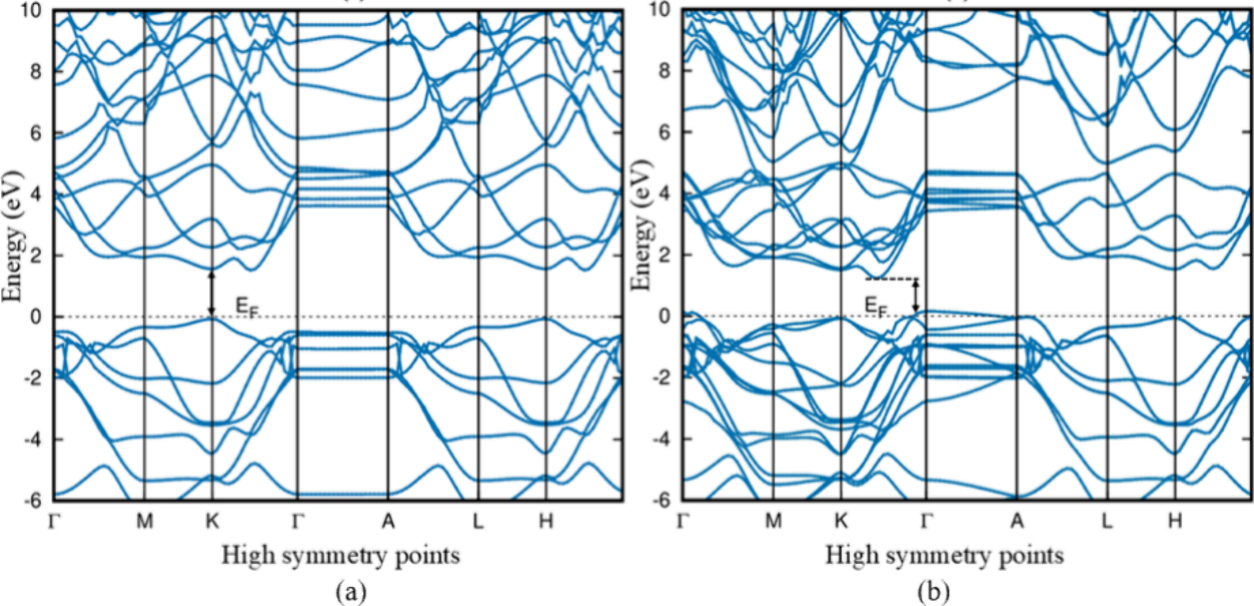
HSE06 band structure for group A polymorphs 1H-MoTe_2_ in (a) and 2H-MoTe_2_ in (b) with direct band and indirect band gaps, respectively.

**Figure 5:**
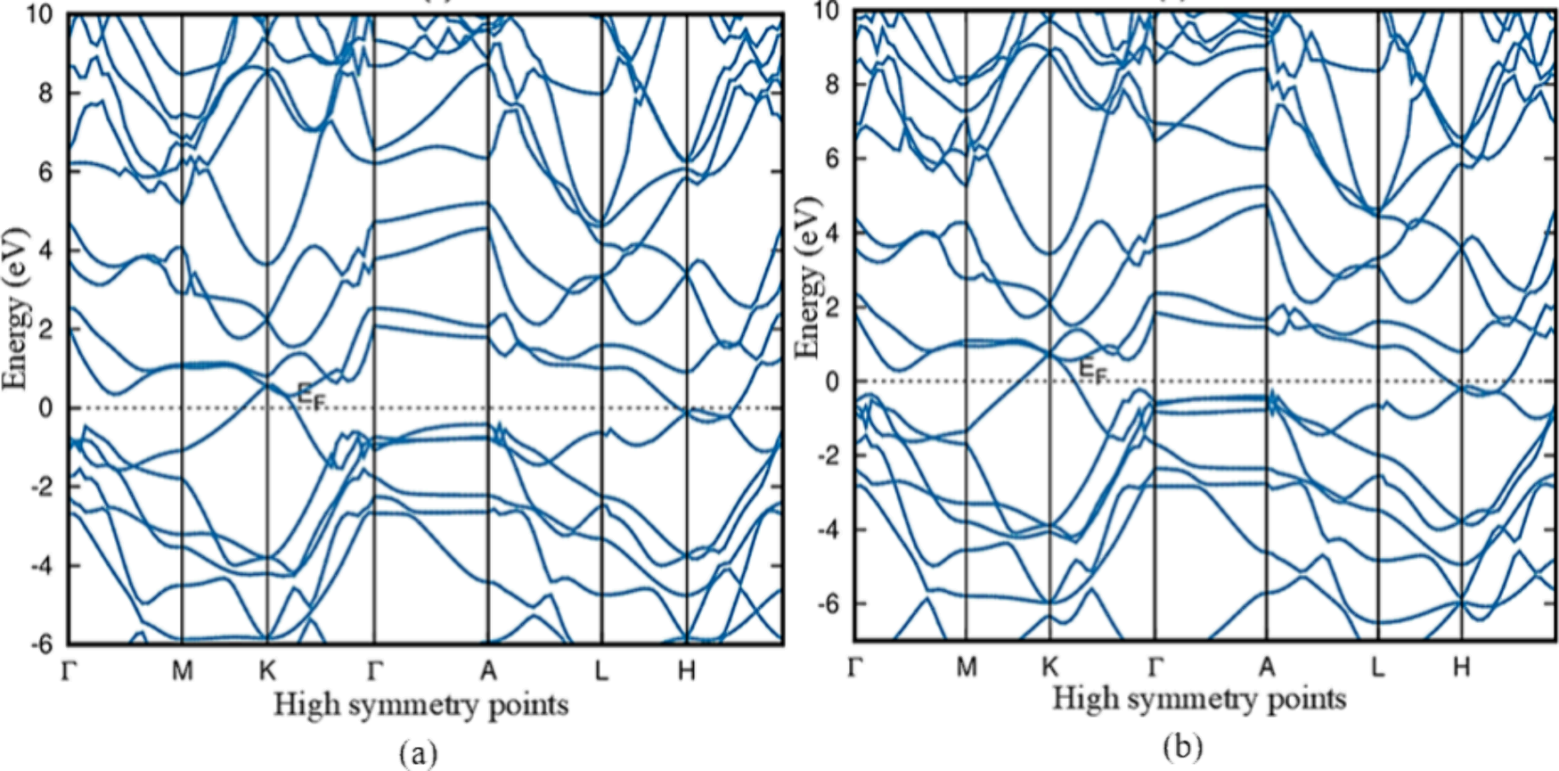
HSE06 band structure of group B polymorphs 1T_1_-MoTe_2_ in (a) and 1T_2_-MoTe_2_ in (b) demonstrates a metallic nature.

The primary difference between 1H-MoTe_2_ and 3H_a_-MoTe_2_ in terms of their electronic band structures lies in the presence of additional bands in 3H_a_. The specific hybridization of these orbitals varies depending on the polymorph, resulting in unique band structures for each MoTe_2._ This difference arises due to its multilayer stacking and interlayer interactions. The other 2H-MoTe_2_, 3H_b_-MoTe_2_, 2T-MoTe_2_, and 2R_1_-MoTe_2_ polymorphs have an indirect bandgap because their CBM is located at the *K*-point while the VBM lies along the K-Γ path in the Brillouin zone.^20^Figure 4 shows the band structure of 1H-MoTe_2_ and 2H-MoTe_2_ polymorphs. The other band structure of group A is given in Figure S1 of the Supporting Information. Except for 4T-MoTe_2_, group A polymorphs have band gaps between 1.01 and 1.5 eV, making them suitable for application in photovoltaic solar cells, photocatalysis, and water-splitting applications. The overlap of the conduction and valence bands of Group B polymorph band structures gives them a metallic nature since they are no longer separated by an energy gap, allowing electrons to travel freely. The band topologies demonstrate the absence of a bandgap, confirming the metallic character of these polymorphs. Figure 5 shows the band structure of 1T_1_-MoTe_2_ and 1T_2_-MoTe_2_, and the other band structure of group B is given in Figure S2 of the Supporting Information on page S2. However, the introduction of an additional layer into the 3T-MoTe_2_ polymorph led to an increase in the number of bands. This suggests that the layering in this polymorph modifies its electronic structure, resulting in a more complex distribution of energy bands while retaining the overall metallic behavior observed in the other group B polymorphs.

In MoTe_2_ polymorphs, the band flatness between the Γ and A points could indeed be influenced by the interlayer spacing in the crystal structure. In materials such as MoTe_2_, the interlayer spacing influences the overlap of atomic orbitals between adjacent layers. If the interlayer distance is large, the wave functions of atoms in one layer have little overlap with those in neighboring layers, leading to flat bands, especially along certain directions in the Brillouin zone. In multilayer materials, the weak van der Waals forces holding the layers together can result in weak interlayer coupling, especially if the layers are not strongly bonded. This weak coupling contributes to the formation of flat bands because the electron hopping between layers is minimized. This flatness could affect the properties of the material in various ways, including the potential for unconventional superconductivity under certain conditions. The band flatness in the band structure may depend on the interlayer spacing of MoTe_2_ polymorphs.^14,20^

In monolayer 1H-MoTe_2_, the effective mass of electrons and holes has been reported as *m*_e_^*^ = 0.56 *m*_0_ and *m*_h_^*^= 0.64 respectively, based on first-principles density functional theory calculations using the HSE06 hybrid functional study of Liu et al.^22^ In bulk 2H-MoTe_2_, the effective masses of electrons and holes are reported as *m*_e_^*^ = 0.70 *m*_0_ and *m*_h_^*^= 0.76, respectively, based on density functional theory calculations with GGA and spin–orbit coupling effects.^23^ This study reports the effective mass of the electrons and holes for the semiconductor polymorphs 1H-MoTe_2_, 2H-MoTe_2_, 3H_a_- MoTe_2_, 3H_b_-MoTe_2_, 2T-MoTe_2_, and 2R_1_-MoTe_2_ which are listed in Table 2.

**Table 2:** Calculated HSE06 and GGA Band Gap Value, the Effective Mass of the Electron (*m*_e_*), the Effective Mass of the Hole (*m*_h_*), and the Band Gap Type of Group-A MoTe_2_ Polymorphs

polymorph	bandgap value in GGA (eV)	band gap value in HSE06 (eV)	effective mass ofelectrons *m*_e_* [*m*_e_] (*m*_0_)	effectivemass ofhole *m*_h_* [*m*_h_]	bandgap type
1H-MoTe_2_	1.188	1.588	0.635	0.777	direct
		1.180^20^			
2H- MoTe_2_	1.007	1.076	0.858	0.826	indirect
		1.050^9^			
		1.080^21^			
2T-MoTe_2_	0.977	1.014	0.915	0.847	indirect
3H_a_-MoTe_2_	1.163	1.498	0.632	0.771	direct
3H_b_- MoTe_2_	1.0085	1.245	0.680	0.768	indirect
3R_1_- MoTe_2_	0.995	1.041	0.749	0.814	indirect

#### 2.2.1 Density of State

**Figure 6:**
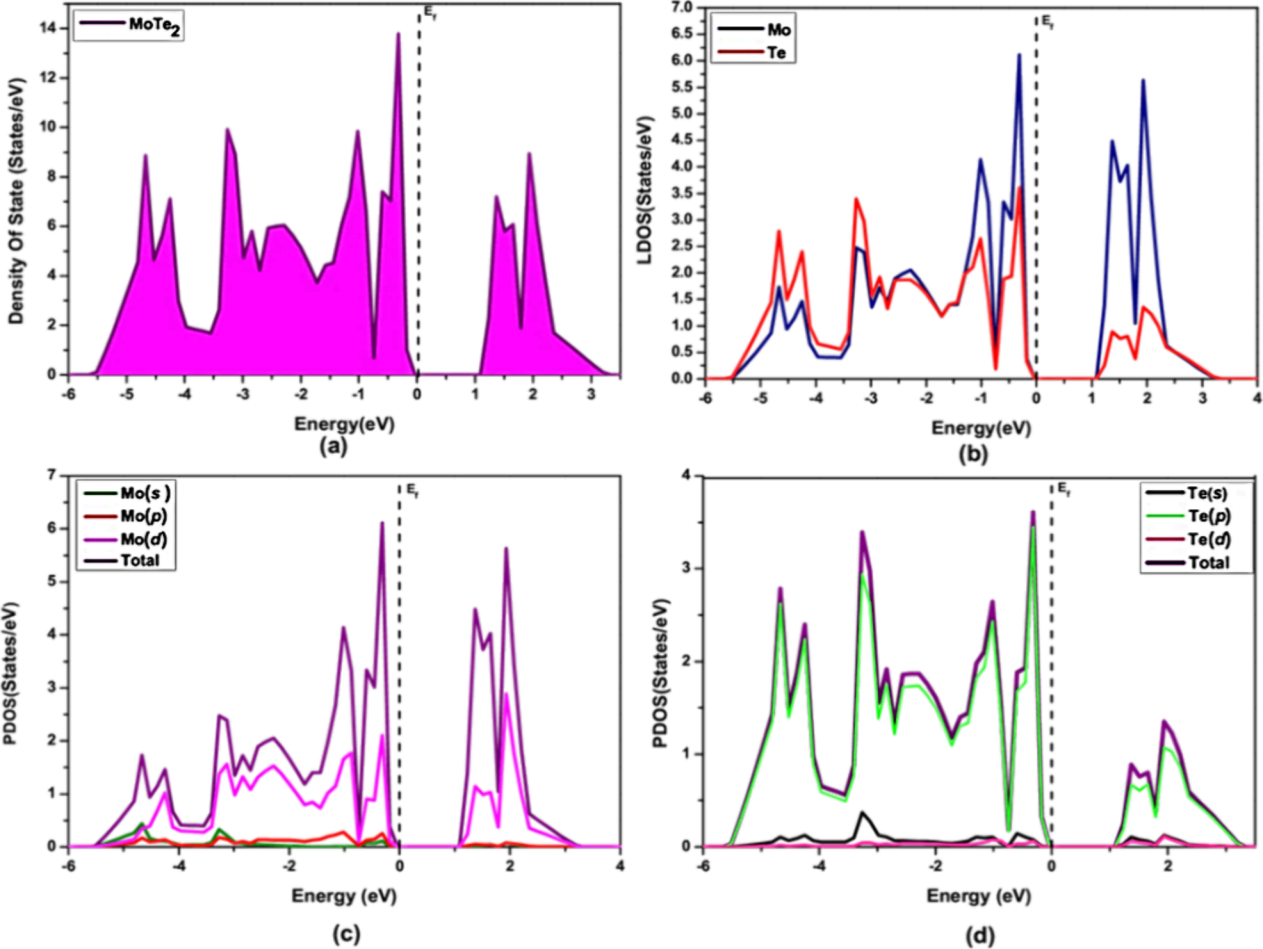
Density of state for 2H- MoTe_2_ in (a), LDOS for 2H- MoTe_2_ in (b), PDOS of Mo in (c), and PDOS of Te in (d).

**Figure 7:**
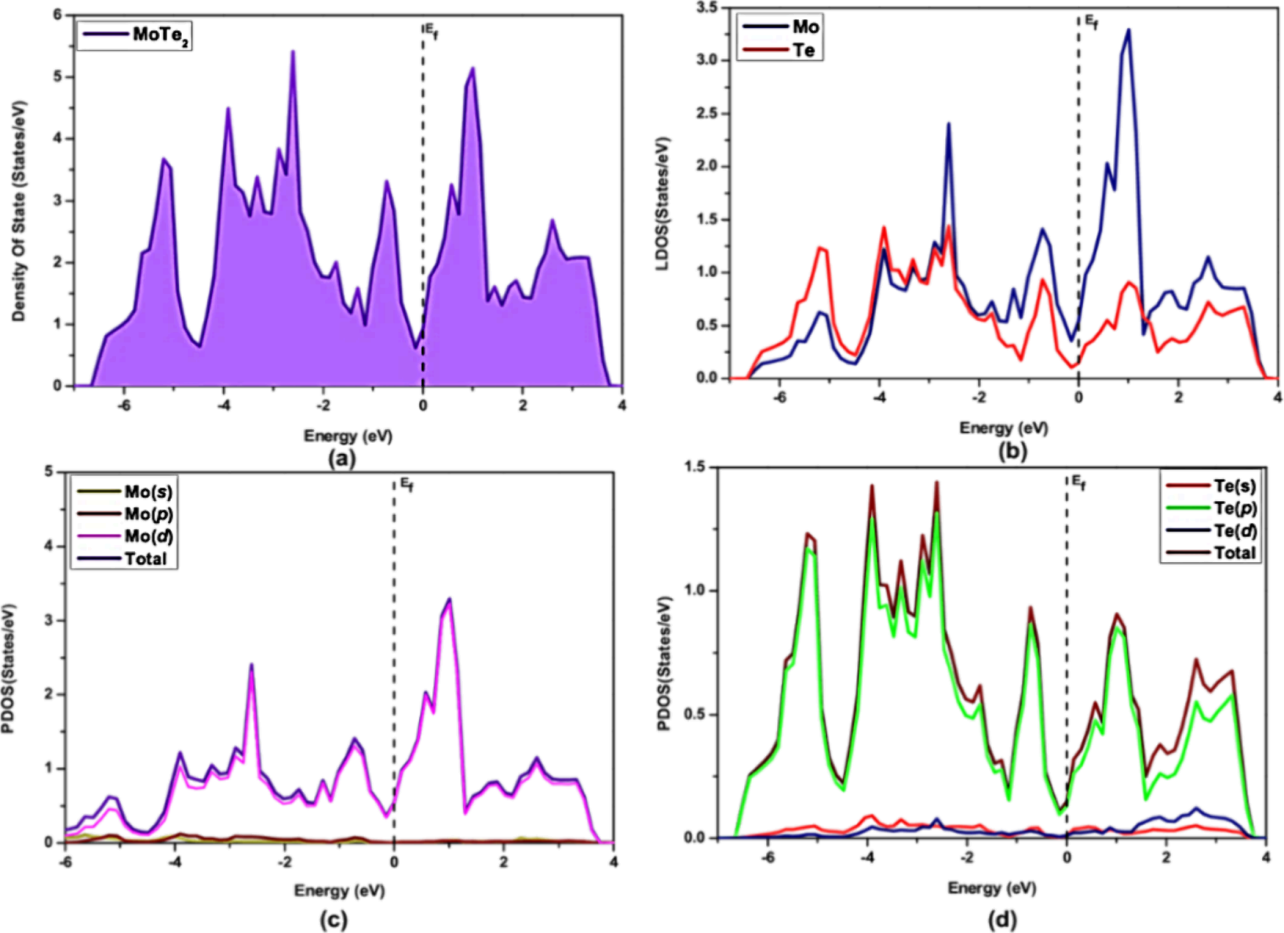
Density of state for 1T_1_- MoTe_2_ in (a), LDOS for 1T_1_-MoTe_2_ in (b), PDOS of Mo in (c), and PDOS of Te in (d).

The density of states (DOS) shows the number of available electronic states at each energy level, while the projected density of states (PDOS) breaks it down by the contribution of individual atomic orbitals to the total DOS.^15,25^ The Fermi level (*E*_F_) is set to zero in the DOS plot. Group A and group B polymorphs exhibit different electronic behaviors, and DOS calculations were performed for all polymorphs. The DOS for 2H-MoTe_2_ shows a semiconducting behavior with an indirect bandgap of approximately 1.07 eV (Figure 6). In 2H-MoTe_2_, the Mo-*d* orbitals contribute mainly to the states below the Fermi level, while the Te-*p* orbitals contribute to the states above the Fermi level. Roldán also analyzed the electronic structure of MoTe_2_ using PDOS and found that the conduction band is mainly composed of Mo-*d* orbitals, while the valence band is mainly composed of Te-*p* orbitals. They further found that the spin–orbit coupling (SOC) effect is significant in MoTe_2_ and leads to a splitting of the valence band maximum (VBM) and conduction band minimum (CBM).^26,27^ 1T_1_-MoTe_2_ is a layered transition metal chalcogenide with a distorted octahedral coordination has a metallic character due to the overlap of the Te-*p* and Mo-*d* orbitals. The electronic structure of 1T_1_-MoTe_2_ can be analyzed using the DOS calculation as shown in Figure 7. The study by Dawson et al. provides a detailed analysis of the electronic and structural properties of MoTe_2_ and WTe_2_ monolayers using DFT calculations. The study finds that both monolayers exhibit metallic behavior with a strong anisotropy in the in-plane directions, and their stability is strongly dependent on the stacking order of the layers. The study also suggests that the stability of the 1T-MoTe_2_ phase is due to the strong hybridization between the metal *d* and chalcogen *p* orbitals.^15,24^

### 2.3 Bonding Nature of Polymorphs

**Figure 8:**
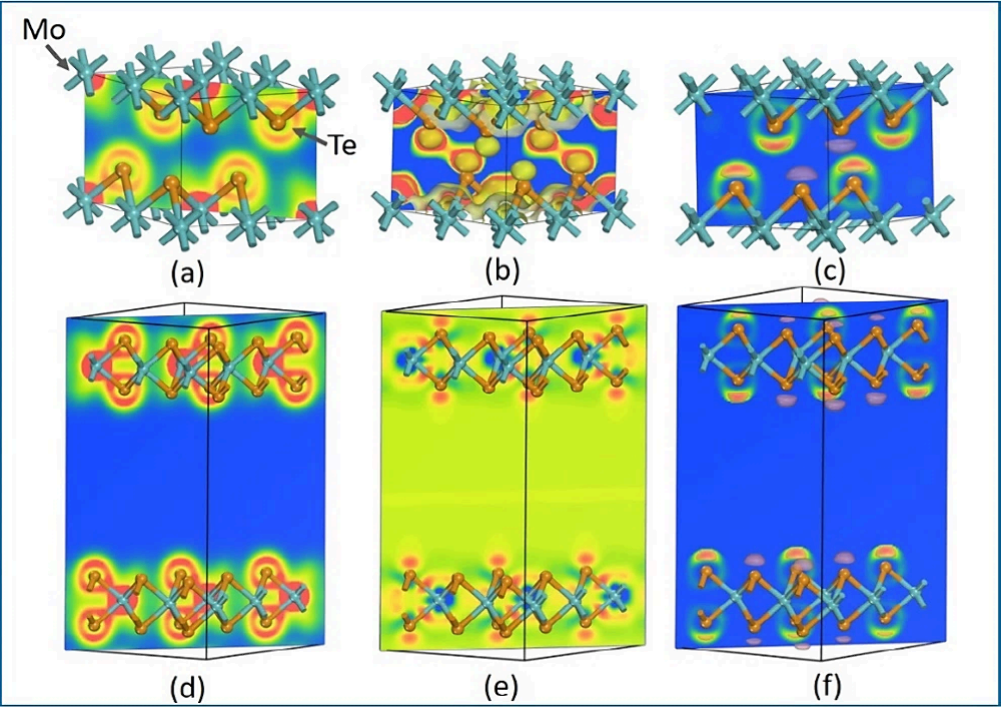
Charge density (a), charge transfer (b), and ELF (c) of trigonal prismatic of MoTe_2_(1T_1_- MoTe_2_) and the charge density (d), charge transfer (e), and ELF (f) of octahedral prismatic of MoTe_2_ (2H- MoTe_2_).

Bonding interactions in polymorphs are described by the projected valence-charge-density distribution. Figure 8 shows the charge density, charge transfer, and electron localization function (ELF) plots for 1T_1_- MoTe_2_ and 2H- MoTe_2_ polymorphs.^18,28^

The trigonal polymorphs of Mo–Te are arranged as shown in Figure 8a, demonstrating typical covalent bonding. In Figure 8d, the charge density of the hexagonal polymorph reveals that Te–Mo–Te bonds form a trigonal pattern, reflecting a similar covalent character. The yellow regions signify a more dispersed position of an anion, whereas the red regions show tightly constrained charge transfer between cations. This covalent nature is evidenced by electron sharing between cations and anions, as depicted in Figure 8b,e.^21,29^Figure 8c,f shows electron localization between Te (like a cap) and Mo atoms in both hexagonal and trigonal forms, where the electron localization is heightened when the ELF value exceeds 0.5, suggesting covalent bonding between Mo and Te.^30−32^ Thus, the charge density, charge transfer, and ELF analyses for both hexagonal and trigonal polymorphs consistently indicate the covalent nature of their bonds.^33^

### 2.4 Dynamical Properties

**Figure 9:**
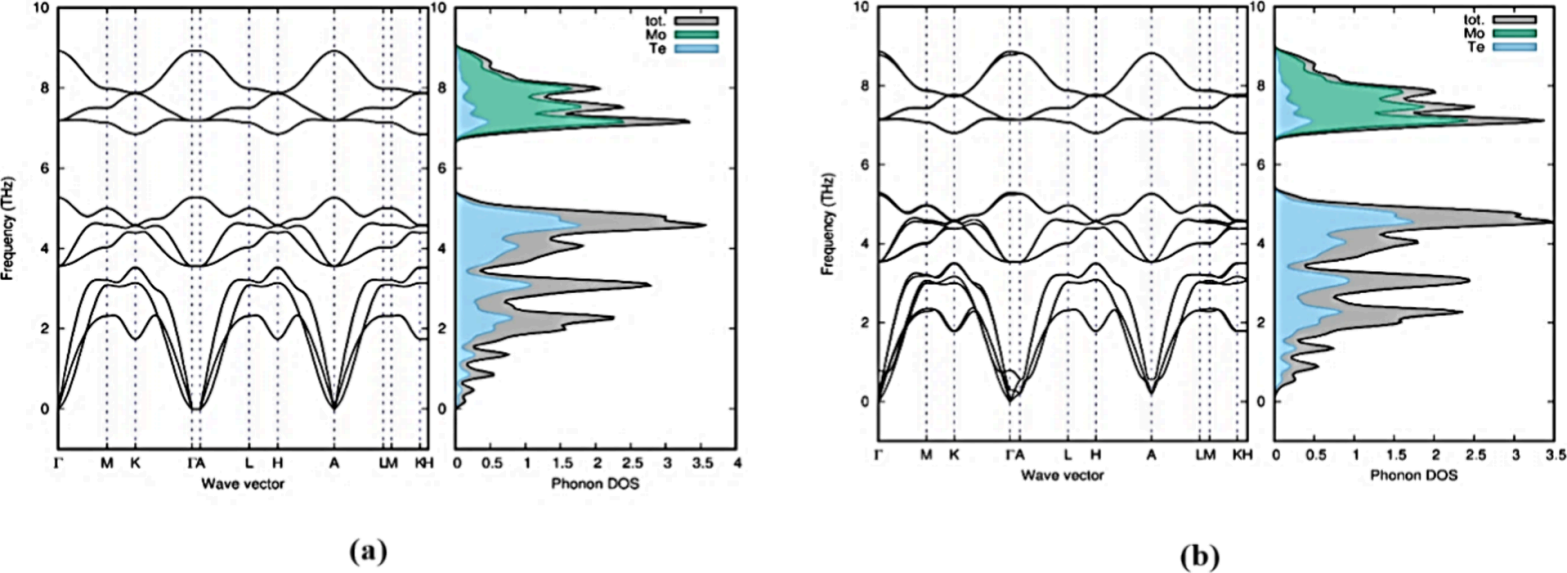
Positive phonon dispersion and phonon density of states for 1H-MoTe_2_ (a) and 2H-MoTe_2_ (b).

Lattice dynamic stability refers to the stability of a crystal structure under small atomic vibrations. It is an essential property that indicates whether a material will remain structurally intact or spontaneously distort. This stability is determined by analyzing phonon frequency vibrational modes within the crystal lattice. If all phonon frequencies are positive, the crystal structure is considered dynamically stable, and it can exist at equilibrium without collapsing or transforming. The lattice dynamical and vibration properties of all the polymorphs are computed by the pre/postprocessing tool of PHONOPY.^34^ The frozen phonon technique has been used to calculate the force constants.^35−37^ In this study, the total phonon density of states (PhDOS) and phonon dispersion curves of all the polymorphs are determined at the equilibrium volume, with the high symmetry direction of the Brillouin zone.^28,38^ In our study, we observed that all of the polymorphs in group A show positive frequencies except 2T-MoTe_2_. The result of this study exhibits the dynamic stability of the six group A polymorphs (1H-MoTe_2_, 2H-MoTe_2_, 3H_a_-MoTe_2_, 4T-MoTe_2_, 3H_b_-MoTe_2_, and 2R_1_-MoTe_2_). 2T-MoTe_2_ polymorph in group A was determined unstable dynamically due to the exhibit of a negative/soft mode of frequencies or a negative eigenvalue. Figure 9 shows the positive phonon frequency and phonon dispersion of 1H-MoTe_2_ and 2H-MoTe_2_. The phonon frequency and phonon dispersion of other polymorphs are given in Figure S3 of the Supporting Information.

**Figure 10:**
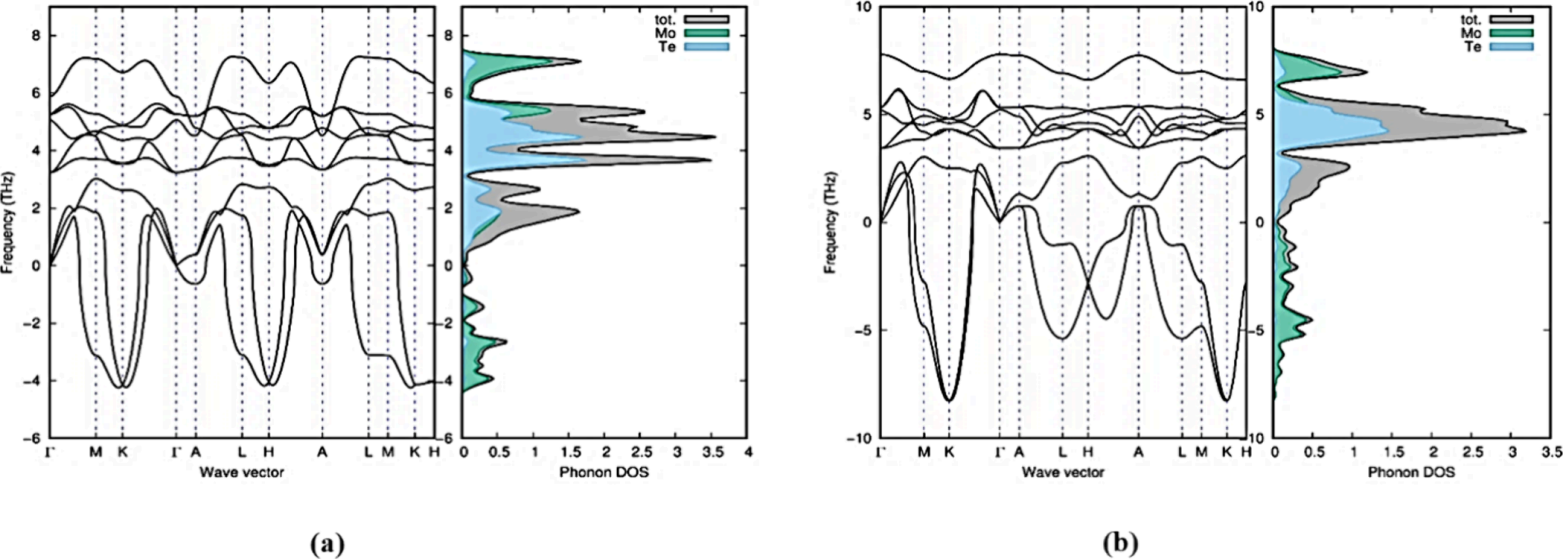
Phonon dispersion and phonon density of states for 1T_1_-MoTe_2_ (a) and 1T_2_-MoTe_2_ (b) polymorphs in group B.

Phonon dispersions of the dynamically stable polymorphs have well-separated optical and acoustical phonons, which are well-suitable for optical properties like photonics, optoelectronics, and medical imaging.^39^ All the stable polymorphs in group A owned the same frequency range of optical modes in the region of 5 and 9 THz. In all the stable polymorphs in group A, the smaller Te atom dominates the lower frequencies, whereas the larger Mo atom dominates the higher frequencies (over 8.5 THz). The stable polymorphs in group A had similar wave vectors for phonon dispersion, demonstrating that temperature and pressure can affect the phase change. As potential energy levels drop close to the equilibrium atomic position, polymorphs become less stable. The soft/negative modes of frequency present in all group B polymorphs exhibit unstable dynamical characteristics for 1T_1_-MoTe_2_, 1T_2_-MoTe_2_, 3T-MoTe_2_, and 2R_2_-MoTe_2_ polymorphs.^40^ For the 1T_1_-MoTe_2_ and 1T_2_-MoTe_2_ polymorphs, Figure 10 displays the unstable phonon dispersion and phonon density of state.^40^Figure S4 of the Supporting Information on page S3 provides information on the other phonon dispersions and phonon densities of states for group B polymorphs.

### 2.5 Mechanical Stability

It is essential to emphasize a complete understanding of the mechanical properties to maximize the potential of polymorphs for a variety of applications. Here, using an elastic constant tool, we estimated the fundamental mechanical characteristics of all polymorphs.^32,42^ The VASPKIT program is used to determine elastic constants by using the “stress-strain” method. Under a small deformation, the linear dependence of the applied strain and the stress component is evaluated based on the tensorial form of the Hooks law. Each stress and strain consists of three tensile and three shears. This 6 × 6 symmetric matrix with 27 different components describes the elastic constant of the crystal. The number of pieces can be naturally decreased by selecting polymorphs with pre-existing symmetry, this stiffness constant *C*_*ij*_ is used to calculate the mechanical properties of the polymorphs. There are six elastic constants for each hexagonal and trigonal polymorph (*C*_11_, *C*_12_, *C*_13_, *C*_33_, *C*_44_, and *C*_66_). The Rhombohedra structure consists of seven elastic constants (*C*_11_, *C*_12_, *C*_13_, *C*_14,_*C*_33_, *C*_44_, and *C*_66_).^32,43,44^

To examine the mechanical stability, the polymorphs have to satisfy the born stability criteria.^44^ In this work 2H-MoTe_2_, 3H_b_-MoTe_2,_ and 2R_1_-MoTe_2_ polymorphs satisfied the stability criteria of elastic constant for hexagonal as well as group B polymorphs satisfied the trigonal stability criteria of elastic constant.^44^ In this study, mechanical properties such as the bulk modulus B, sheared modulus G, Young’s modulus E and Poisson’s ratio ν are calculated for all the polymorphs using the Voigt(V)-Reuss(R)-Hill(H) approach.^45^Tables 3 and 4 show the bulk modulus B, sheared modulus *G*, Young’s modulus *E*, Poisson’s ratio ν, and Born stability criteria of the elastic constant for group A and group B respectively.

**Table 3:** Calculated Single-Crystal Elastic Constants *C_ij_* (in GPa), Born Criteria, the Bulk Modulus B (in GPa), Shear Modulus *G* (in GPa), Poisson’s Ratio Ν, Young’s Modulus *E* (in GPa), and Pugh Ratio (*B*/*G*) of the Group A Polymorph

polymorph	1H -MoTe_2_	3H_a_-MoTe_2_	3H_b_-MoTe_2_	2H-MoTe_2_	4T-MoTe_2_	2T-MoTe_2_	2R_1_-MoTe_2_
crystal system	hexagonal	hexagonal	hexagonal	hexagonal	trigonal	trigonal	rhombohedra
*C*_11_	51.02	81.58	117.91	123.43	101.73	80.84	125.07
				101^9^			
*C*_12_	11.65	18.97	27.33	32.32	20.50	16.39	29.95
				32^9^			
*C*_13_	0.48	0.88	2.65	11.13	0.93	0.87	8.15
*C*_14_	0	0	0	0	- 0.01	0.05	1.76
*C*_33_	1.25	2.26	7.41	25.86	2.20	2.38	20.00
*C*_44_	–0.12	–0.07	4.06	3.49	–0.80	–0.54	7.80
*C*_66_	19.68	31.30	45.28	45.55	40.61	32.22	47.56
Born	No	No	Yes	Yes	No	No	Yes
*B*	7.75	12.61	20.69	32.82	15.00	12.30	29.28
*G*	4.80	7.84	15.78	16.18	8.64	7.16	20.32
*E*	11.95	19.50	37.75	41.69	21.74	17.99	49.51
Ν	0.24	0.24	0.19	0.28	0.25	0.25	0.21
*B*/*G*	1.61	1.61	1.31	2.03	1.74	1.72	1.44

**Table 4:** Calculated Single-crystal Elastic Constants *C*_*ij*_ (in GPa), Born Criteria the Bulk Modulus *B* (in GPa), Shear Modulus *G* (in GPa), Poisson’s Ratio Ν, Young’s Modulus *E* (in GPa), and Pugh Ratio (*B*/*G*) of the Group B Polymorphs

polymorph	1T_1_-MoTe_2_	IT_2_- MoTe_2_	2R_2_-MoTe_2_	3T-MoTe_2_
crystal system	trigonal	trigonal	rhombohedra	trigonal
*C*_11_	132.39	141.13	135.24	150.66
*C*_12_	6.97	14.77	5.41	9.95
*C*_13_	26.95	42.12	17.68	19.03
*C*_14_	0	0	1.99	2.83
*C*_33_	38.23	52.16	30.49	56.79
*C*_44_	8.32	20.81	3.01	31.84
*C*_66_	62.71	63.18	64.91	70.35
Born	Yes	Yes	Yes	Yes
*B*	41.53	54.56	35.41	46.42
*E*	58.30	75.91	48.34	99.15
*G*	23.02	29.93	18.99	43.33
Ν	0.26	0.26	0.27	0.14
*B*/*G*	1.80	1.82	1.86	1.06

In Table 3, 2H -MoTe_2_, 3H_b_-MoTe_2_, and 2R_1_-MoTe_2_ polymorphs from group A are identified as mechanically stable, satisfying the Born stability criteria. other polymorphs from group A failed to meet these criteria, indicating mechanical instability. In group A, 1H-MoTe_2_, 4T-MoTe_2_, and 3H_a_-MoTe_2_ polymorphs have stable phonons, however, because of their unstable mechanical properties, these are classified as metastable polymorphs. The elastic constants, including bulk modulus *B*, shear modulus *G*, Young’s modulus *E*, Poisson’s ratio ν, and the Born stability criteria for Group B polymorphs, are detailed in Table 4. Although group B polymorphs meet the mechanical stability requirements, they are dynamically unstable, which poses a challenge for the experimental synthesis. In group A, the 2R_1_-MoTe_2_ polymorph exhibits the highest *C*_11_ value, while the 1H-MoTe_2_ polymorph has the lowest. As shown in Tables 3 and 4, all polymorphs display *C*_11_ values that exceed *C*_33_, indicating that the *x*–*y* plane is stiffer than the *z*-direction. Furthermore, each polymorph has a *C*_12_ value greater than *C*_13_, suggesting that tension is more effectively distributed along the *x*- and *z*-axes than along the *y*-axis. Due to their relatively low *C*_44_ values, the 1H-MoTe_2_, 4T-MoTe_2_, and 3H_a_-MoTe_2_ polymorphs in Group A are more susceptible to shear deformation. Further, group B MoTe_2_ polymorphs exhibit higher bulk modulus and shear modulus values (Table 2), highlighting the increased hardness of the 2R_1_-MoTe_2_ and 3T-MoTe_2_ polymorphs. The Pugh’s ratio (*B*/*G*) provides additional insights, where a higher ratio (>1.75) suggests ductility, while a lower ratio (<1.75) indicates brittleness.^41^ In group A, the 2H-MoTe_2_ polymorph stands out with a high Pugh’s ratio, indicating ductile behavior. Except for 2H-MoTe_2_, the Pugh’s ratio for all other Group A polymorphs is below 1.75, classifying them as brittle in nature. Conversely, in group B, only the 3T-MoTe_2_ polymorph exhibits a Pugh’s ratio below 1.75, indicating brittleness, while all other group B polymorphs exceed this threshold, signifying ductility.

**Figure 11:**
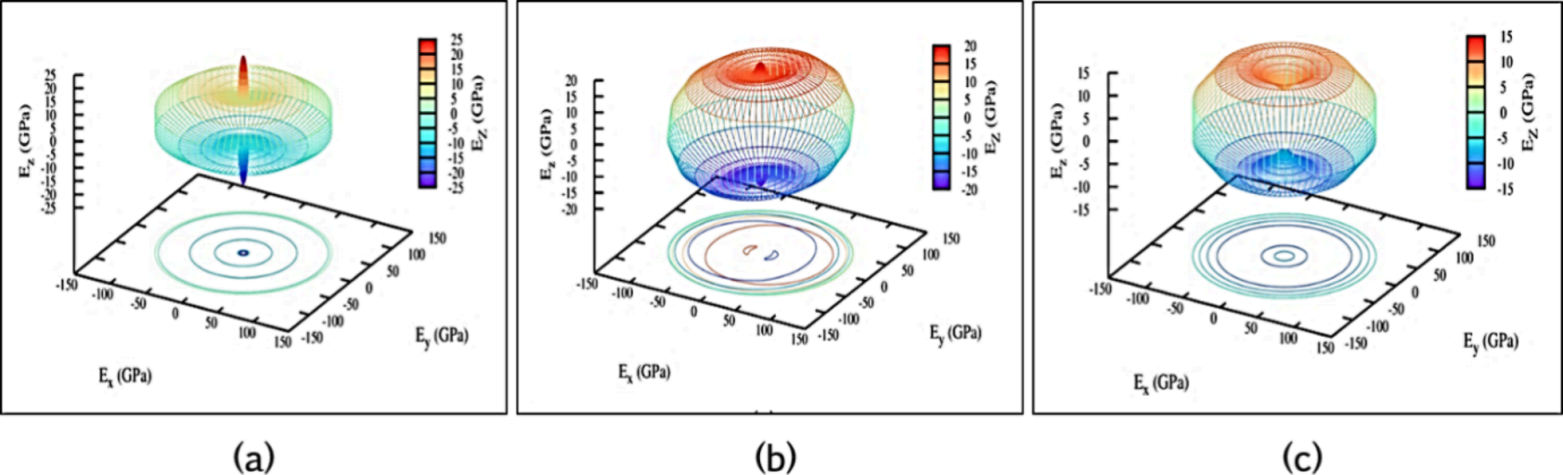
3D plot of Young’s modulus (*E*) for stable polymorphs 2H-MoTe_2_ in (a), 3H_b_-MoTe_2_ in (b), and 2R_1_-MoTe_2_ in (c).

Poisson’s ratio (ν) is another criterion for distinguishing between brittle and ductile materials, with values greater than 0.26 indicating ductility. Among the studied polymorphs, the 2H-MoTe_2_ in group A and 2R_2_-MoTe_2_ in group B exhibit higher Poisson’s ratios, suggesting a more ductile nature compared to other polymorphs. With the exception of 2H-MoTe_2_, all group A polymorphs have Poisson’s ratios below 0.26, reflecting their inherent weakness. The presence of a positive Young’s modulus (*E*) indicates that atoms in stable polymorphs exhibit compressibility. In this study, all group A polymorphs display positive values for Young’s modulus, with the 2R_1_-MoTe_2_ polymorph showing a particularly high value, suggesting greater atomic compressibility compared to other polymorphs. By evaluation of the physical properties and anisotropy of *E* and ν, this investigation establishes the isotropic characteristics of mechanically stable polymorphs. Young’s modulus, derived from elastic compliance constants, also reveals the orientation-dependent behavior of the polymorphs.^32,46^ For isotropic materials, Young’s modulus surface should ideally exhibit a spherical shape. Figure 11 presents a 3D plot of Young’s modulus for the dynamically and mechanically stable polymorphs 2H-MoTe_2_, 3H_b_-MoTe_2_, and 2R_1_-MoTe_2_. Among these, 2R_1_-MoTe_2_ shows the highest Young’s modulus, whereas 2H-MoTe_2_ exhibits the lowest within group A.^38,43^

### 2.6 Thermodynamical Properties

**Figure 12:**
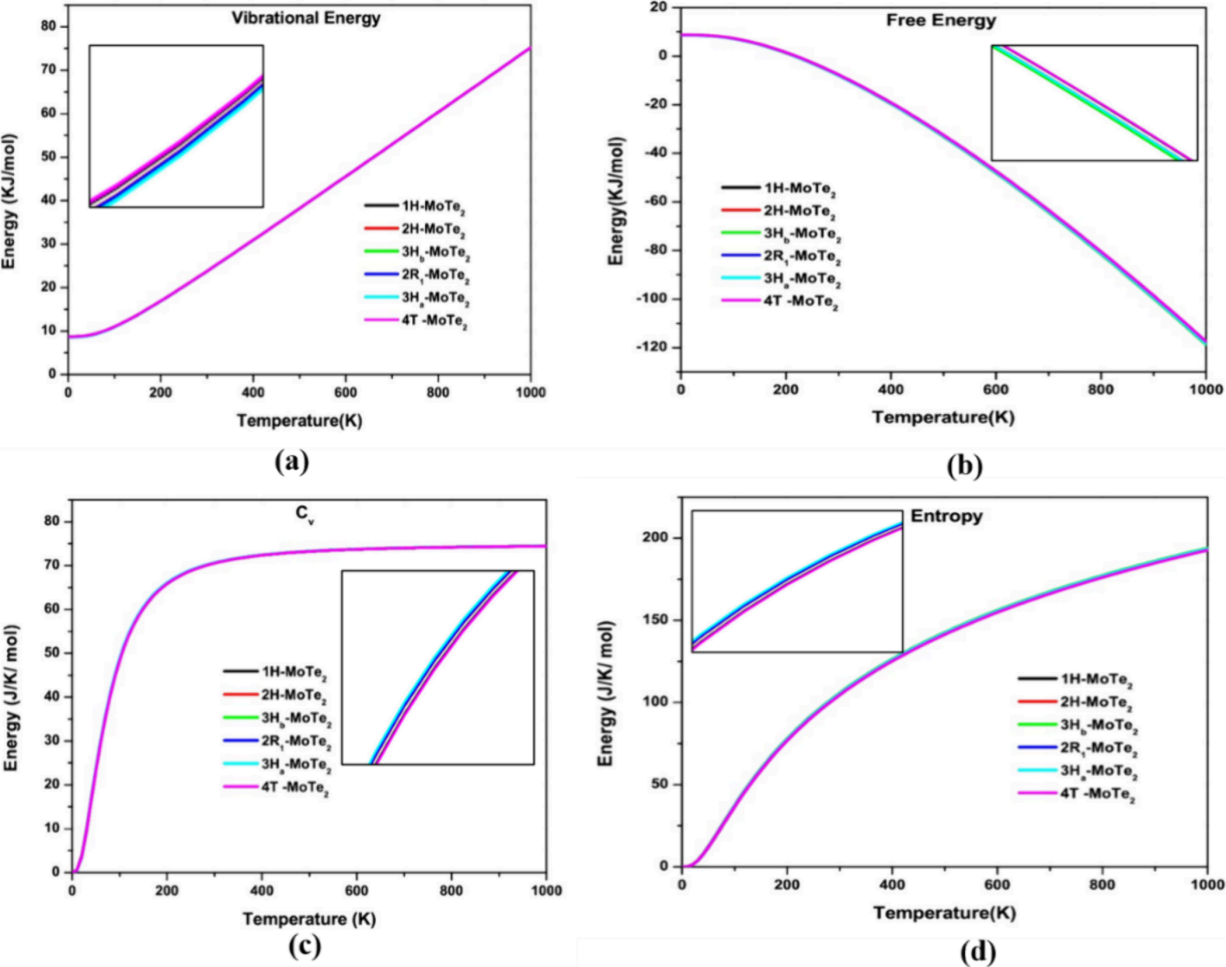
Vibrational free energy “*F*” (a) internal energy “*E*” (b), specific heat at constant volume “*C*_v_” (c), and Entropy “*S*” (d) as a function of temperature for dynamic stable MoTe_2_ polymorphs.

The thermodynamic analysis is used to predict the temperature-dependent behavior of dynamically stable polymorphs.^34^ Thermodynamic parameters that vary with temperature, including specific heat at a constant volume (*C*_v_), entropy (*S*), internal energy (*E*), and vibrational free energy (*F*), are presented in Figure 12. Figure 12a,b shows the exponential increase in vibrational energy and the exponential decrease in free energy.^38,36^Figure 12c shows that the specific heat at constant volume (*C*_v_) increases linearly with temperature, indicating that all dynamically stable polymorphs follow the classical Dulong-Petit law at high temperatures, where the specific heat approaches a constant value. At lower temperatures, the behavior transitions to follow Debye’s T^3^ law, which describes the temperature dependence of specific heat in solids at low temperatures, reflecting the contribution of acoustic phonons to thermal properties.^12^ All of the dynamically stable polymorphs increase the entropy as the temperature rises by the third rule of thermodynamics. Energy as a function of temperature in Figure 12d shows the maximum value reaches 178 J/K/mol at 1000 K, for all dynamically stable polymorphs. Our observations for various temperatures follow the two key laws of thermodynamics, suggesting that all of the dynamic polymorphs under investigation are thermodynamically stable even for wider pressure ranges. Although no theoretical or experimental results have been found, it is challenging to compare the results.

### 2.7 IR and Raman Spectra

#### 2.7.1 Raman Spectra

Raman spectroscopy is a powerful technique for characterizing phonon properties at the zone center in crystalline materials.^6^ Raman spectra are calculated for the dynamically stable polymorphs (1H-MoTe_2_, 2H-MoTe_2_, 3H_a_-MoTe_2_, 4T-MoTe_2_, 3H_b_-MoTe_2_, and 2R_1_-MoTe_2_) to understand the vibrational behavior. Table 5 shows the Raman active modes for the dynamically stable polymorphs. The 1H-MoTe_2,_ polymorph shows that the *A*_1_^′^ mode involves out-of-plane vibrations where Mo and Te atoms oscillate perpendicularly to the basal plane of the layer. Zhang et al., discuss the E′ mode (in-plane) around 235 cm^–1^ and the *A*_1_^′^ mode (out-of-plane) near 170 cm^–1^.

**Table 5:** Raman Active Modes and IR Active Modes for the Dynamically Stable Polymorphs

polymorph	Raman activemode (cm^–1^)	IR activemode (cm^–1^)
1H-MoTe_2_	*E*’: 233	*A*_2_^″^: 288
	^2^*E*″: 117	^2^*E*’: 233
	*A*_1_^′^: 171	
2H- MoTe_2_	^2^*E*_2g_: 233	*A*_2u_: 287
	^2^*E*_1g_: 117	^2^*E*_1u_: 233
	*A*_1g_: 171, 171^48^	
3H_a_- MoTe_2_	^2^*E*’: 117, 233	^2^*A*_2_^″^: 288
	^2^*A*_1_^′^: 3, 171, 288	^4^*E*’: 117, 233
	^2^*E*″: 6, 117, 233	
3H_b_- MoTe_2_	^2^*E*_2g_: 11, 233	*A*_2u_: 285
	^2^*E*_1g_: 117	^2^*E*_1u_: 233
	*A*_1g_: 171	
4T- MoTe_2_	^2^*E*_g_: 117,233	*A*_2u_: 171,287,288
	*A*_1g_: 171,287,288	^2^*E*_u_: 117,233
2R_1_- MoTe_2_	^2^*E*: 171,233	*A*_2u_: 285
	^2^*A*_1_: 117,288	^2^*E*_1u_: 233

**Figure 13:**
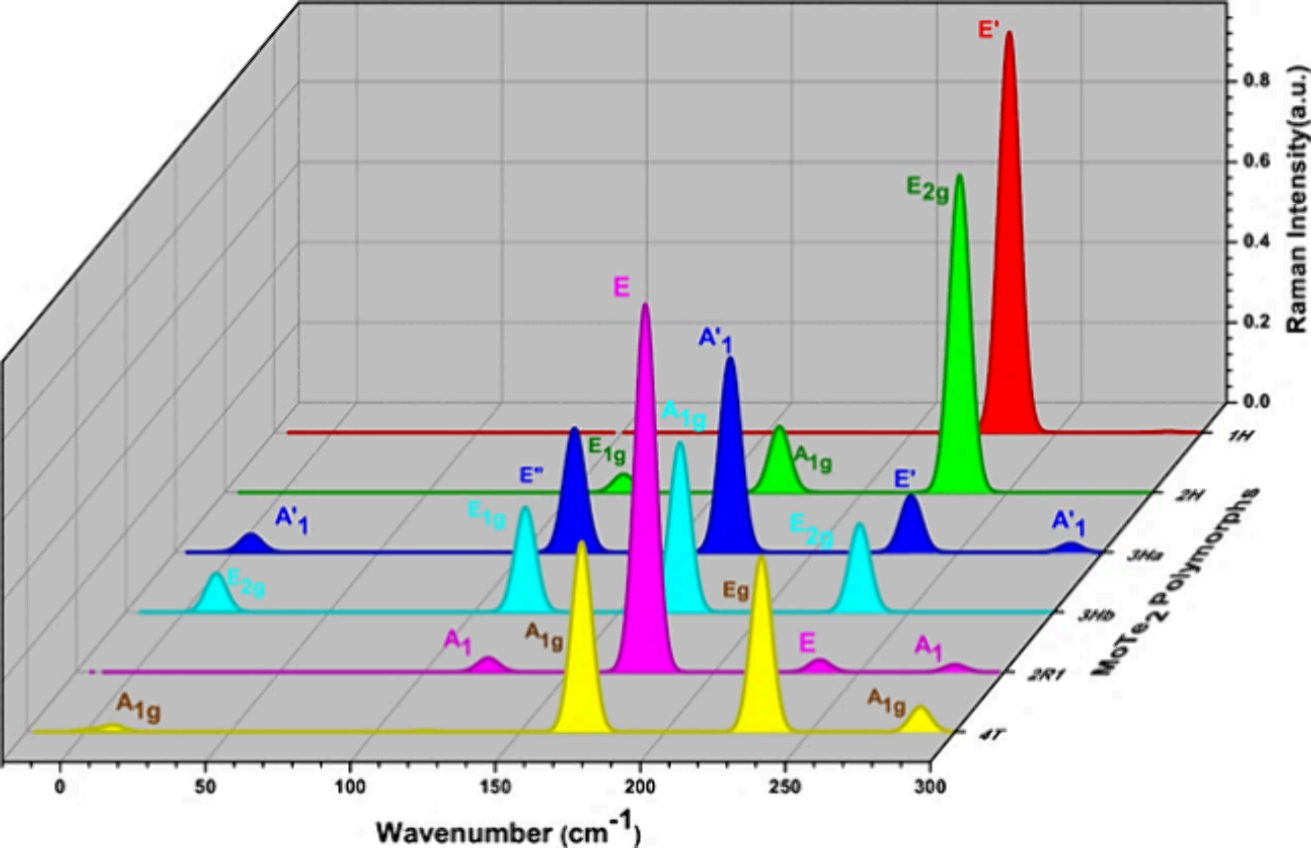
Raman intensity as a function of wavenumber for dynamically stable polymorphs.

These modes are typically used to characterize monolayer MoTe_2_ and confirm its structural and thickness attributes.^6^ In the 2H-MoTe_2_ polymorph, the *A*_1g_ mode involves out-of-plane vibrations and is typically observed around 171 cm^–1^_._*E*_2g_ modes typically appear around 233 cm^–1^ for 2H-MoTe_2_. Its frequency and intensity provide insights into the structural integrity and layer interactions and can be used to detect strain effects. Particularly out of the plane mode for all polymorphs occur at 233 cm^–1^ (Figure 13) and in-plane modes occur at the same point, 171 cm^–1^, which is well coincides with the experimental value.^48^ This demonstrates that despite the differences in the polymorphs, the mode of vibration appears at the same distance for all the polymorphs.^49^ Low-frequency modes can arise in multilayer 3H_a_-MoTe_2_ and 3H_b_-MoTe_2_ due to interlayer shear and breathing vibrations. These modes occur below 50 cm^–1^ and are useful for determining the layer number and studying interlayer coupling.

#### 2.7.2 IR Spectrum

**Figure 14:**
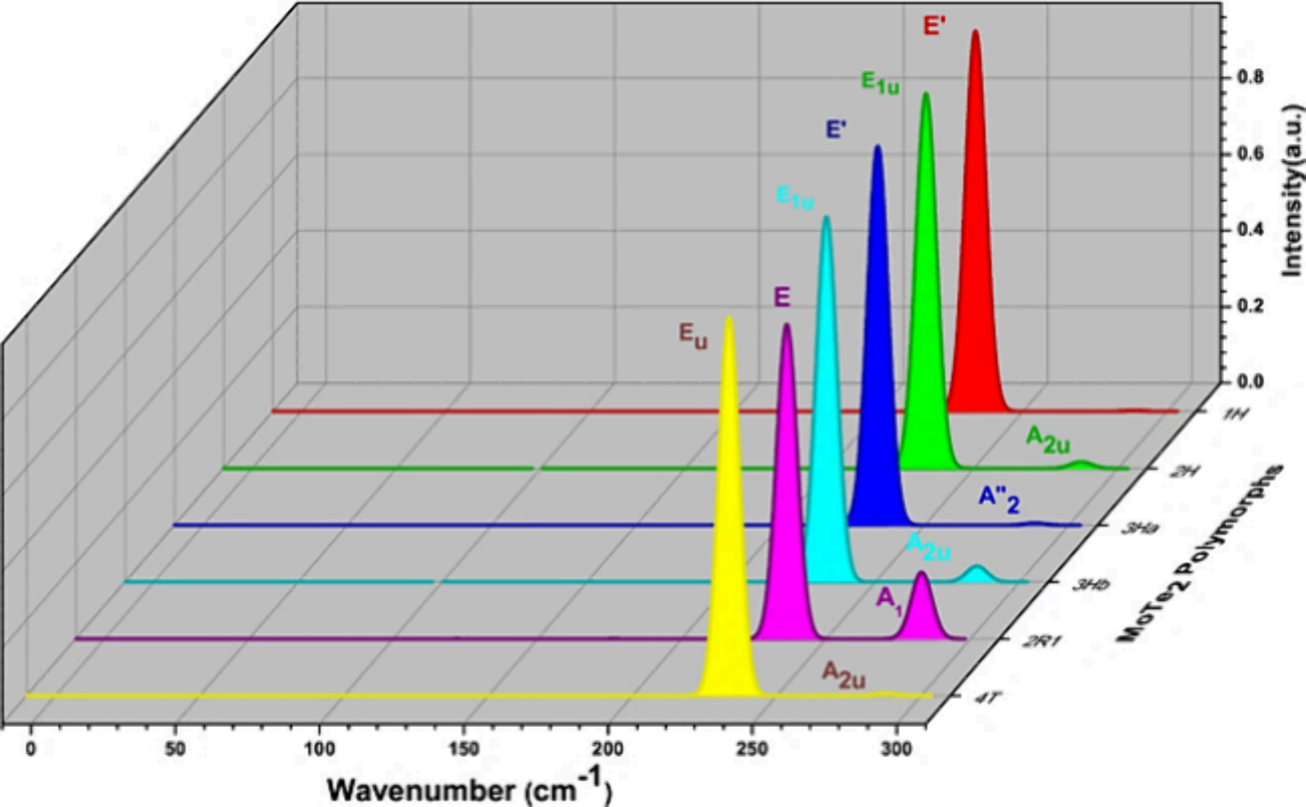
IR intensity as a function of wavenumber for dynamically stable MoTe_2_ polymorphs.

The stable phonon polymorphs involved in the IR spectra are investigated and the corresponding modes of representation are evaluated in this paper.^50^Table 5 shows the IR active modes for the dynamically stable polymorphs. In our observation the phonon shows two IR modes are active, one is in the plane (*E*’) and another one is out of the plane for all stable polymorphs.^51^ All of the phonon-stable polymorphs in Figure 14 show the in-plane mode at the same wavenumber value of 233 cm^–1^. All polymorphs show the strong mode of the peak which dominates in plane mode.^52^ The *A*_2_^″^ IR mode of 1H-MoTe_2_ and 3H_a_-MoTe_2_ involves out-of-plane vibrations of the Te atoms concerning the Mo layer. This mode results in perpendicular oscillations to the basal plane, which are typical for layered structures. The frequency varies from 285 to 288 cm^–1^ depending on the number of layers and different structures of MoTe_2_.^48^

## 3 Conclusions

In conclusion, the stability analysis of MoTe_2_ polymorphs reveals that group A structures (1H-MoTe_2_, 2H-MoTe_2_, 3H_a_-MoTe_2_, 3H_b_-MoTe_2_, 2T-MoTe_2_, 4T-MoTe_2_, 2R_1_-MoTe_2_) exhibit lower total energies, indicating higher stability compared to group B (1T_1_-MoTe_2_, 1T_2_-MoTe_2_, 3T- MoTe_2_ and 2R_2_-MoTe_2_). The electronic structure analysis highlights the semiconducting nature of group A and the metallic behavior of group B MoTe_2_ polymorphs. The bandgap and effective mass values, particularly for 1H-MoTe_2_ and 2H-MoTe_2_, show promise for applications in photovoltaics and photocatalysis, while the metallic properties of group B polymorphs suggest potential in conductive applications. The DOS and PDOS analyses reveal that 2H-MoTe_2_ exhibits semiconducting behavior with distinct orbital contributions from Mo-*d* and Te-*p* states, while 1T_1_-MoTe_2_ displays metallic character due to the strong overlap of these orbitals. These findings underscore the influence of orbital hybridization and stacking on the electronic properties of the MoTe_2_ polymorphs. The charge density, charge transfer, and ELF analyses consistently confirm the covalent bonding character between the Mo and Te atoms in both hexagonal and trigonal MoTe_2_ polymorphs. The result of this study confirms the dynamic stability of the six polymorphs 1H-MoTe_2_, 2H-MoTe_2_, 3H_a_-MoTe_2_, 4T-MoTe_2_, 3H_b_-MoTe_2_, and 2R_1_-MoTe_2_. This study analyzes the mechanical properties of MoTe_2_ polymorphs, identifying stable and metastable forms. 2R_1_-MoTe_2_, 3H_b_-MoTe_2_, and 2H-MoTe_2_ display ductility and exhibit strong mechanical properties. The Raman and IR spectral analyses of MoTe_2_ polymorphs highlight consistent vibrational modes across different structures. Phonon and elastic tensor studies reveal that the polymorphs 1H-MoTe_2_, 3H_a_-MoTe_2_, and 4T-MoTe_2_ are metastable, while polymorphs 2H-MoTe_2_, 3H_b_-MoTe_2_, and 2R_1_-MoTe_2_ exhibit dynamic and mechanical stability. This comprehensive study highlights the application potential of these stable MoTe_2_ polymorphs in the electronic, mechanical, and optoelectronic fields.

## 4 Method of Calculation

In the periodic density functional theory, the VASP (Vienna Ab Initio Simulation Package) code is used for the initial optimization.^45,54^ Generally, the interaction between the core and valence electrons is described by the projector-augmented wave (PAW) approach.^47,55,56^ In this study, with the use of the Pardew-Burke-Ernzerhof (PBE) exchange-correlation, the structure of each polymorph is initially optimized. To obtain PBE level optimal structure, the DFT/vdw-df2 approach is applied.^57^ From the optimized structure, an energy volume curve was computed to determine the lowest energy. Structure-related parameters can only be applied to forecast dependability for large E-cuts, we employed a 550 eV energy cutoff.^58^ The screened hybrid function, developed by Heyd, Scuseria, and Ernzerhof, was used to identify the electrical characteristics of polymorphs that are optimized at the PBE level (HSE06).^59^ The polymorphs employed a Monkhorst-pack 2 × 2 × 1 as *k*-mesh for structural optimization. By calculating the periodic Kohn–Sham equation on ten *k*-points along each direction of high symmetry of the irreducible part of the first Brillouin zone, bands of the polymorphs are calculated.^60^ Using the CASTEP (Cambridge Sequential Total Energy Package) program, investigations of charge density, charge transfer, and electron localization function (ELF) were carried out, which aided in understanding the nature of the bonds and interactions between the MoTe_2_ polymorphs.^61^ For the supercells of the polymorphs, a suitable supercell mode is performed, and the phonon dispersion and phonon density of the state are calculated using PHONOPY software.^60^ The VASP code is used to determine the supercell’s force constant. To get the matrices to create a force constant, each atom in the binary system is moved by a finite displacement of 0.007 in the *x*, *y*, and *z* directions. After determining the force constant, we build a dynamic matrix in the Brillouin zone for various q vectors. To obtain the phonon frequency eigenvalues and mode eigenvectors, the dynamic matrices are solved. Table S2 of the accompanying information on page S4 lists a suitable supercell mode and Monkhorst-pack gird. By comparing imaginary and real polymorphic modes, we examined the dynamical stability of each polymorph is examined. For the investigated polymorphs, thermal parameters, such as heat capacity, free energy, and system entropy, are derived. The dynamical stability of all polymorphs is checked by observing imaginary and real modes of polymorphs. Thermal properties are obtained for the studied polymorphs, including heat capacity, free energy, and entropy of the system. In this work, single crystal elastic constants are computed to understand the mechanical stability of all polymorphs. The stress tensor is generated after a series of strains (−0.015, −0.010, −0.005, 0.000, 0.005, 0.010, and 0.015) have been applied to each crystal system. By linear fitting of the stress–strain curve, VASPKIT is used to calculate the elastic constants. All of the polymorphs of MoTe_2_ have their Raman and IR spectra obtained using the CASTEP software.^53^ For the CASTEP computations, we used an optimized VASP structure to obtain a precise result.^61^

## Supporting Information Available

Minimum energy, electronic structure, phonon dispersion relation of polymorphs, *k*-grid, and supercell size of MoTe_2_ polymorphs (PDF)
