# Supplementary material for: MoTe2 Polymorphs: A DFT Approach to Structural, Electronic, Mechanical and Vibrational Properties
Source: ACS Omega. 2025 Mar 24;10(13):13515–28. doi: 10.1021/acsomega.5c00226 (PMC11983220; doi:10.1021/acsomega.5c00226)
Supplement: Supplementary file 1 — ao5c00226_si_001.pdf [file ao5c00226_si_001.pdf]

$MoTe_2$ Polymorphs: A DFT Approach to Structural, Electronic, Mechanical and Vibrational Properties

Lathifa Banu S[a], Kanimozhi Balakrishnan[b], Vasu Veerapandy[b], Nalini Vajeeston[c] and Ponniah Vajeeston[c*]

[a] *Department of Physics, Sethu Institute of Technology, Kariyapatti, Virudhunagar - 626115, Tamil Nadu, India.*

[b] *Department of Computational Physics, School of Physics, Madurai Kamaraj University, Madurai 625021, Palkalai Nagar, Tamil Nadu, India.*

[c] *Department of Chemistry, Center for Materials Science and Nanotechnology, University of Oslo, Oslo 0371, Norway.*

1. Structure and the Stability of $MoTe_2$ Polymorphs

Table S1. The calculated minimum energy(E) with respect to the volume(Å^3) of optimized $MoSe_2$ polymorphs and the formation energy of optimized $MoSe_2$ polymorphs

Polymorph	Volume(Å^3)	Minimum Energy (eV/f.u.)
**Group-A**		
1H-$MoTe_2$	166.20	-20.04
2H-$MoTe_2$	64.12	-20.06
3H$_a$-$MoTe_2$	112.04	-20.05
3H$_b$-$MoTe_2$	62.09	-20.07
2T-$MoTe_2$	103.95	-20.07
4T-$MoTe_2$	81.86	-20.08
2R$_1$-$MoTe_2$	60.93	-20.08
**Group-B**		
1T$_1$-$MoTe_2$	58.60	-19.33
1T$_2$-$MoTe_2$	59.14	-19.41
3T-$MoTe_2$	57.94	-19.44
2R$_2$-$MoTe_2$	59.86	-19.40

[*] Corresponding author
Email address: vajeeston.ponniah@kjemi.uio.no

2. Electronic structure

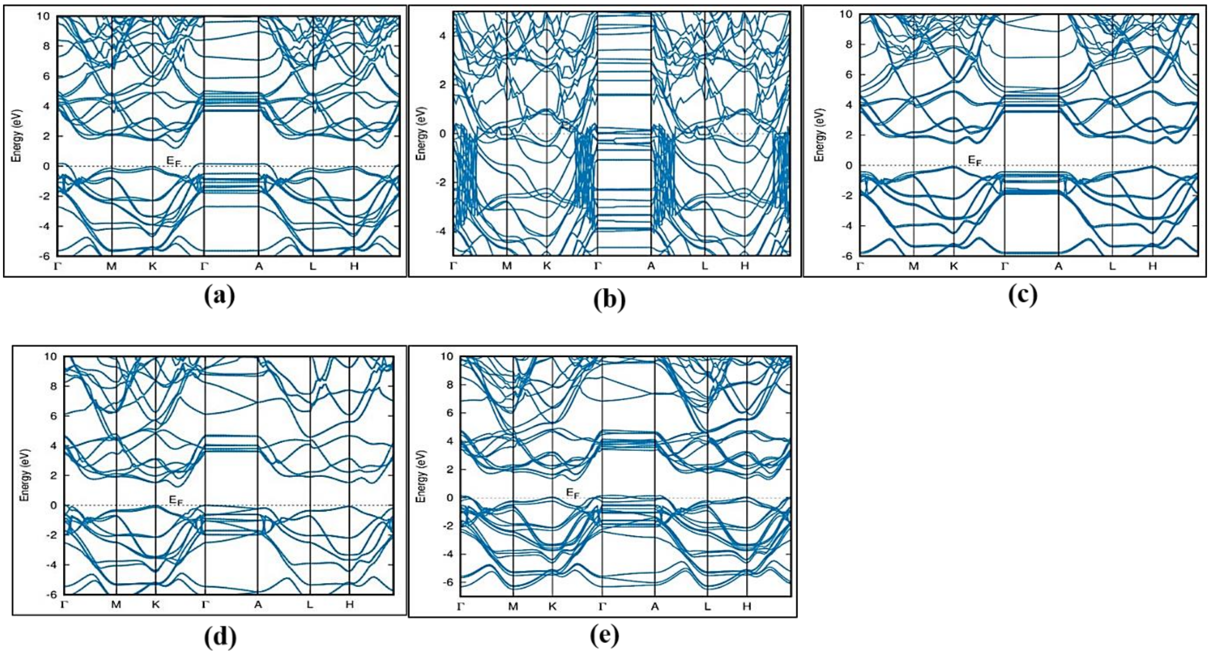

Figure S1. HSE06 band structure for group A ($3H_a$-$MoTe_2$in (a), 4T-$MoTe_2$ in (b) $3H_b$-$MoTe_2$in (c), 2T- $MoTe_2$(d), and $2R_1$-$MoTe_2$ in (e)). We see that the group A polymorphs are semiconductors with a bandgap between 1.07 and 1.58, except for 4T-$MoTe_2$

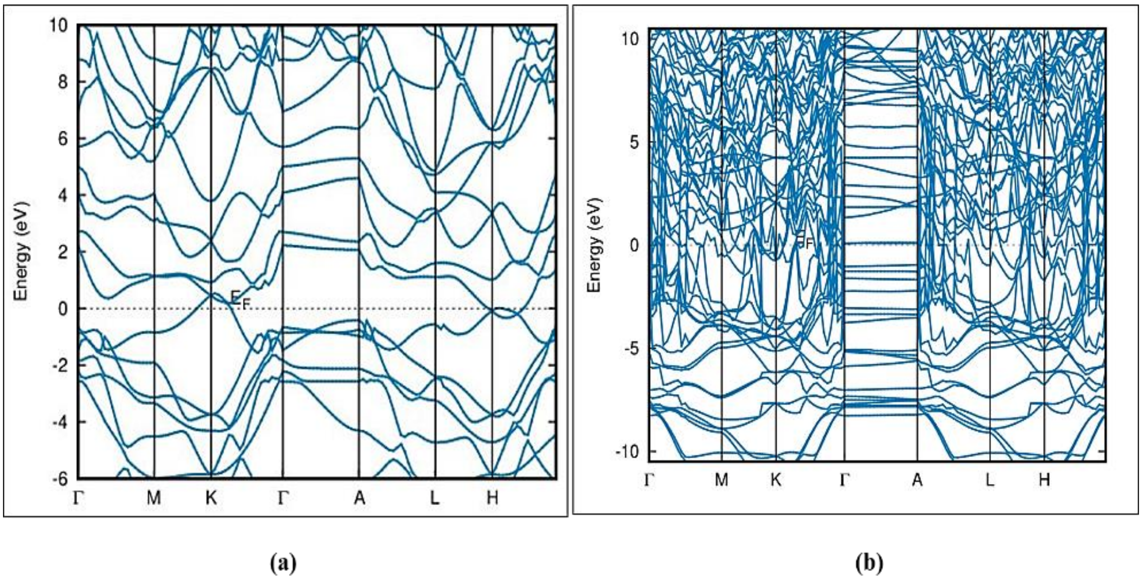

Figure S2. HSE06 band structure for group B ($2R_2$-$MoTe_2$ in (a) and 3T-$MoTe_2$in (b)).

3. Dynamical properties

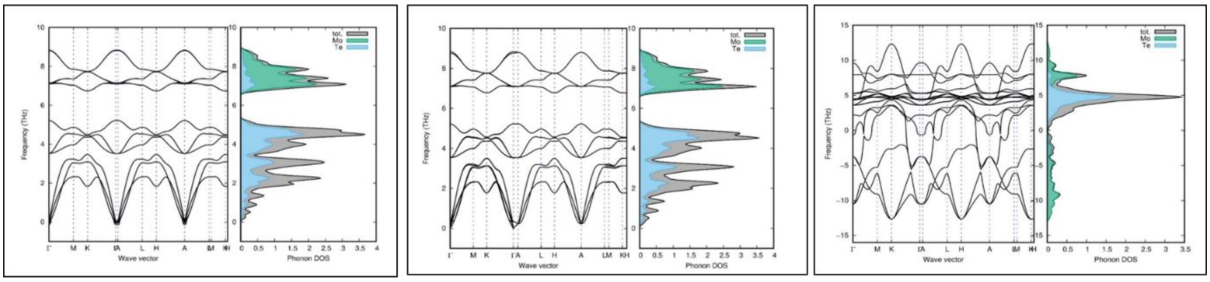

(a) (b) (c)

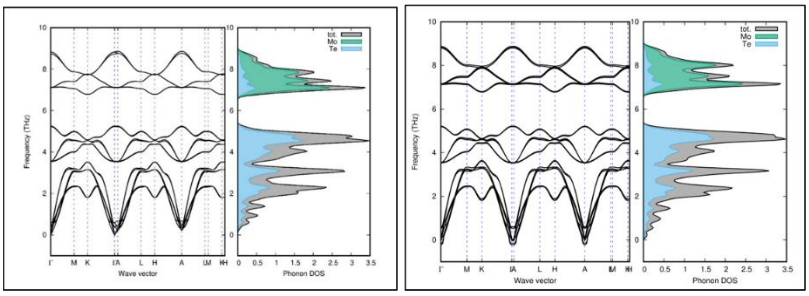

(d) (e)

Figure S3. Phonon dispersion and phonon density of states for $3H_a$ -$MoTe_2$ (a), $3H_b$-$MoTe_2$ (b), 2T- $MoTe_2$(c), $2R_1$-$MoTe_2$ (d) and 4T- $MoTe_2$(e) polymorphs in group A.

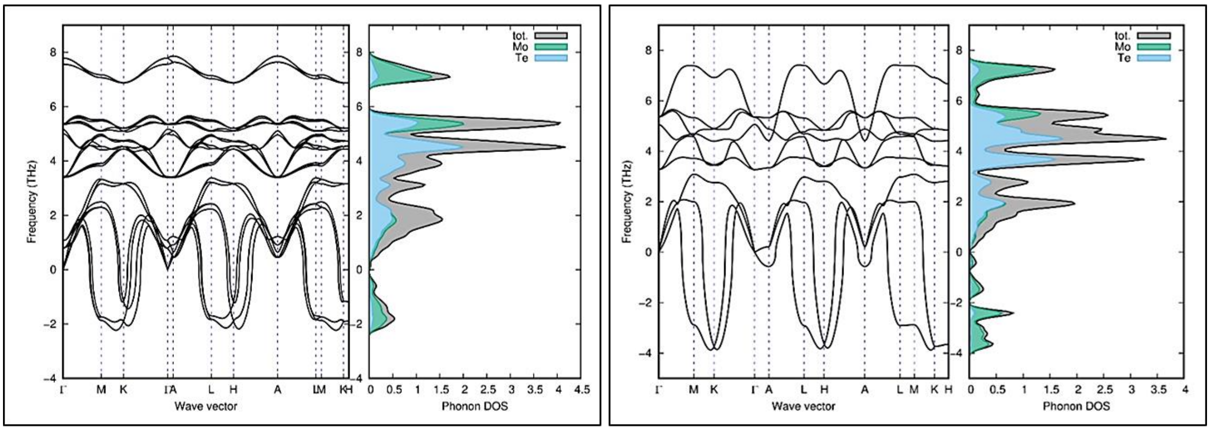

(a) (b)

Figure S4. Phonon dispersion and phonon density of states for 3T-$MoTe_2$ (a), and $2R_2$-$MoTe_2$ (b) polymorphs in group B. All the group B polymorphs are holds negative frequencies, which means dynamically unstable

4. METHOD OF CALCULATION

Table S2. Used k-grid size for the HSE calculation and supercell size for the phonon calculation for the involved polymorphs

**Polymorphs**	**k-grid**	**Supercell size**	**Polymorphs**	**k-grid**	**Supercell size**
**Group-A**			**Group-B**		
1H-$MoTe_2$	8 ×8 × 8	4 × 4 × 1	$1T_1$-$MoTe_2$	12 × 12 ×12	4× 4 × 2
2H-$MoTe_2$	8 × 8 × 8	4 × 4 × 1	$1T_2$-$MoTe_2$	8 × 8 × 8	4 × 4 × 2
$3H_a$-$MoTe_2$	8 × 8 × 8	4 × 4 × 1	3T-$MoTe_2$	12 × 12 ×12	3 × 4 × 1
$3H_b$-$MoTe_2$	8 × 8 × 8	4 × 4 × 1	$2R_2$-$MoTe_2$	8 × 8 × 8	3 × 4 × 1
2T-$MoTe_2$	8 × 8 × 8	4 × 4 × 1			
4T-$MoTe_2$	8 × 8 × 8	3 × 4 × 1			
$2R_1$-$MoTe_2$	8 × 8 × 8	3 ×4 × 1			
